# Caffeic acid and chlorogenic acid mediate the ADPN-AMPK-PPARα pathway to improve fatty liver and production performance in laying hens

**DOI:** 10.1186/s40104-025-01175-z

**Published:** 2025-04-03

**Authors:** Wenjie Tian, Gerard Bryan Gonzales, Hao Wang, Youyou Yang, Chaohua Tang, Qingyu Zhao, Junmin Zhang, Huiyan Zhang, Yuchang Qin

**Affiliations:** 1 State Key Laboratory of Animal Nutrition and Feeding, Institute of Animal Sciences, Chinese Academy of Agricultural Sciences, Beijing, 100193 China; 2 Department of Public Health and Primary Care, Faculty of Medicine and Health Sciences, Ghent University, Ghent, Belgium

**Keywords:** Absorptivity, Caffeic acid, Chlorogenic acid, Fatty liver, Laying hens

## Abstract

**Background:**

Caffeic acid (CA) and its derivative, chlorogenic acid (CGA), have shown promise in preventing and alleviating fatty liver disease. CA, compared to CGA, has much lower production costs and higher bioavailability, making it a potentially superior feed additive. However, the efficacy, mechanistic differences, and comparative impacts of CA and CGA on fatty liver disease in laying hens remain unclear. This study aimed to evaluate and compare the effects of CA and CGA on production performance, egg quality, and fatty liver disease in laying hens.

**Results:**

A total of 1,440 61-week-old Hyline Brown laying hens were randomly divided into 8 groups and fed diets supplemented with basal diet, 25, 50, 100 and 200 mg/kg of CA, and 100, 200 and 400 mg/kg of CGA (CON, CA25, CA50, CA100, CA200, CGA100, CGA200 and CGA400, respectively) for 12 weeks. Both CA and CGA improved production performance and egg quality, while reducing markers of hepatic damage and lipid accumulation. CA and CGA significantly decreased TG, TC, and LDL-C levels and increased T-SOD activity. Transcriptomic and proteomic analyses revealed that CA and CGA reduced hepatic lipid accumulation through downregulation of lipid biosynthesis-related genes (*ACLY*, *ACACA*, *FASN*, and *SCD1*) and enhanced lipid transport and oxidation genes (*FABPs*, *CD36*, *CPT1A*, *ACOX1*, and *SCP2*). Of note, low-dose CA25 exhibited equivalent efficacy to the higher dose CGA100 group in alleviating fatty liver conditions. Mechanistically, CA and CGA alleviated lipid accumulation via activation of the ADPN-AMPK-PPARα signaling pathway.

**Conclusions:**

This study demonstrates that dietary CA and CGA effectively improve laying performance, egg quality, and hepatic lipid metabolism in laying hens, with CA potentially being more economical and efficient. Transcriptomic and proteomic evidence highlight shared mechanisms between CA25 and CGA100. These findings provide a foundation for CA and CGA as therapeutic agents for fatty liver disease and related metabolic diseases in hens, and also offer insights into the targeted modification of CGA (including the isomer of CGA) into CA, thereby providing novel strategies for the efficient utilization of CGA.

**Graphical Abstract:**

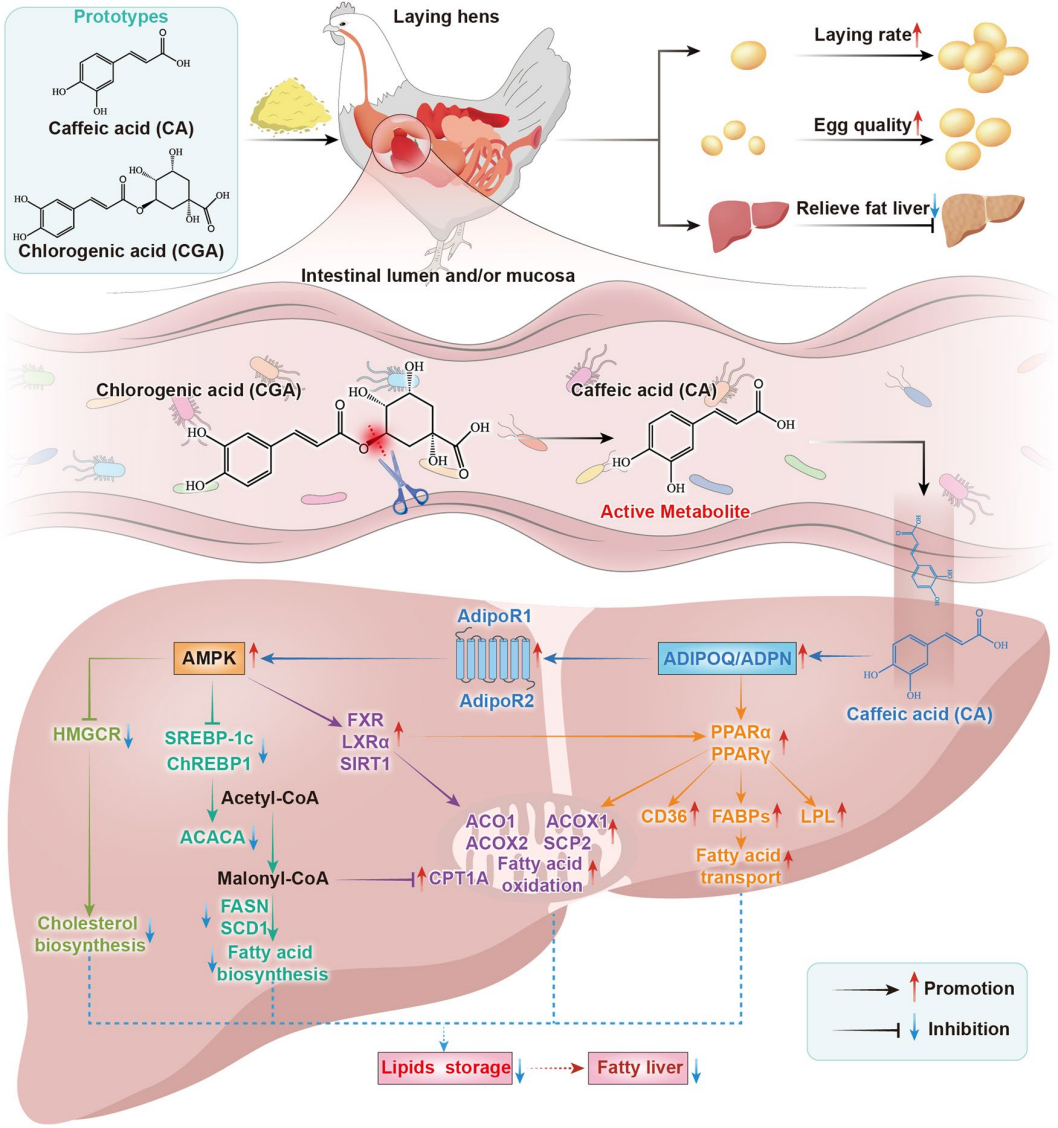

**Supplementary Information:**

The online version contains supplementary material available at 10.1186/s40104-025-01175-z.

## Highlights

(1) Dietary CA and CGA improve fatty liver, laying performance and egg quality.

(2) Lower dose of CA25 achieves the equivalent improvement as CGA100 or CGA200.

(3) CA and CGA mediate the ADPN-AMPK-PPARα pathway to alleviate fatty liver.

**Supplementary Information:**

The online version contains supplementary material available at 10.1186/s40104-025-01175-z.

## Introduction

As laying hens gradually enter the later laying stages, their production performance and egg quality begin to decline [1–3], accompanied by significant health challenges. Among these, fatty liver disease emerges as a critical concern, characterized by excessive hepatic lipid deposition, coupled with organ aging and poor health. This disease is the major cause of non-infectious death in caged laying hens and has enormous economic ramifications for the poultry industry [4–7]. Fatty liver is characterized by the pathological accumulation of hepatic and abdominal lipid, manifesting as lipid metabolism disorder, hepatic steatosis, and inflammatory reactions [8–10], largely driven by genetics, nutrition, environment, and hormones [11–14].

Fatty liver in mammals is usually caused by an increase in liver fatty acid uptake and synthesis, coupled with an inability to timely transport or oxidatively decompose these fats resulting in excessive fat accumulation in hepatocytes and eventually leading to hepatic steatosis [15]. This process can further develop into severe fatty liver when uncontrolled [14]. Unlike mammals however, avian species rely heavily on the liver for lipogenesis, with 90%–95% of de novo fatty acid synthesis occurring in this organ [16, 17]. Thus, reducing hepatic lipid storage is an effective intervention strategy to alleviate fatty liver in laying hens.

Caffeic acid (CA) and chlorogenic acid (CGA) are abundant in coffee beans, Eucommia leaves, honeysuckle and tea, and are also one of the major active ingredients of these plants [18, 19]. These compounds are widely studied in the alleviation of metabolic diseases such as obesity and metabolically associated fatty liver disease (MAFLD) due to their excellent lipid-lowering effect [20]. Mechanistically, both CA and CGA exhibit protective effects against liver steatosis [21–24], oxidative stress [25, 26], inflammation [27], and fibrosis [28] in mammals. Furthermore, studies indicate that both CA and CGA exert anti-lipogenesis effect by activating the AMPK signaling pathway [29–31]. Unfortunately, the low absorptivity of CGA is a primary factor limiting the efficient utilization among various kinds of diseases at present [32]. Studies have shown that most of the CA is absorbed into the blood, while only about one third of the CGA is absorbed [33, 34]. Meanwhile, the efficiency of CGA utilization largely depends on its metabolism by gut microflora in rats [32], with CA as the primary metabolite, accounting for 57.4% of CGA intake [32, 35].

At present, CA production has a much lower cost advantage and whether CA has equivalent beneficial effect as CGA is unknown in laying hens. Based on these studies, the pharmacological action of CGA is perhaps largely determined by how much CGA is enzymatically decomposed into CA and enters the liver to exert biological effects. However, these conjectures need to be further verified. Meanwhile, the beneficial effects and pharmacological mechanisms of CA and CGA on fatty liver in laying hens have not yet been documented.

Therefore, we hypothesized that both CA and CGA can improve fatty liver disease and production performance of laying hens, but the utilization efficiency of CA is higher and feed cost is more economical. To explore this hypothesis, the effect of various concentrations of CA and CGA was tested and the mechanisms by which CA and CGA may alleviate fatty liver and improve production performance in the aged laying hens were explored. The findings of this study can provide a theoretical basis and research foundation for the application of CA and CGA to alleviate fatty liver in laying hens.

## Materials and methods

### Animals and experimental design

A total of 1,440 61-week-old Hyline Brown laying hens were randomly divided into 8 groups, with each group consisting of 10 replicates and 18 hens per replicate. The hens were fed with diets supplemented with 0 (basal diet), 25, 50, 100 and 200 mg/kg of caffeic acid (CA), or 100, 200 and 400 mg/kg of chlorogenic acid (CGA) (CON, CA25, CA50, CA100, CA200, CGA100, CGA200 and CGA400 groups, respectively) for 12 weeks. CA and CGA were purchased from Shanxi Bolin Biotechnology Co., Ltd. (Shanxi, China) (purity > 98%). The CA and CGA supplemental doses were based on references reported in other animals [36–38]. Feed and water were available ad libitum throughout the experiment, which followed a lighting schedule of 16 h of light and 8 h of darkness during the whole experimental period. The composition and nutrient contents of the basal diet are presented in Table 1. All diets were formulated to meet or exceed the estimated nutrient requirements for laying hens as recommended by the NRC (1994).

**Table 1:** Composition and nutrient contents of basal diet

Ingredient, %	Content	Calculated nutrition levels^c^, %	Content
Corn (8.5% CP)	62.2	Crude protein	15.63
Soybean meal (46% CP)	23.2	Metabolizable energy, kcal/kg	2,635
Wheat bran	3.796	Calcium	3.84
Limestone	9	Total phosphorus	0.48
CaHPO_4_	0.85	Available phosphorus + phytase	0.32
Zeolite powder	0.169	Lysine	0.79
Salt	0.3	Methionine	0.39
Choline chloride (50%)	0.1	Threonine	0.59
DL-Methionine (99%)	0.14	Tryptophan	0.18
Phytase (≥ 10,000 U/g)	0.02	Methionine + cystine	0.66
Mineral premix^a^	0.025		
Vitamin premix^b^	0.2		
Total	100		

^a^The mineral premix provided the following per kg of diets: Mn 54 g, Fe 36 g, Cu 5 g, I 0.8 g, Zn 45 g, Se 0.18 g

^b^The vitamin premix provided the following per kg of diets: vitamin A 38,000,000 IU, vitamin D_3_ 15,000,000 IU, vitamin E 60 g, vitamin K_3_ 7 g, vitamin B_1_ 6 g, vitamin B_2_ 30 g, vitamin B_6_ 15 g, vitamin B_12_ 0.05 g, pantothenic acid 40 g, niacin 80 g, folic acid 1.5 g, biotin 0.2 g

^c^Nutrient levels were calculated values, which based on the formula data of the basal diet

### Sample collection and procedures

At the end of the 12-week feeding period, one hen was randomly selected from each replicate per treatment group after a 12-h feed deprivation. The body weight was recorded, and blood was collected from the brachial vein and centrifuged at 4 °C to obtain plasma, which was then stored at −80 °C for further analysis. Another tube of anticoagulant blood (whole blood) was immediately sent to the laboratory for routine blood tests. The hens were sacrificed by exsanguination following cervical dislocation. Liver tissues were immediately removed and weighed, and then the morphology was photographed and observed. The liver specimens were fixed using 4% paraformaldehyde solution for the production of pathological sections. Parts of the livers were then rapidly frozen in liquid nitrogen and stored at −80 °C for further analysis.

### Measurements of laying performance and egg quality

Throughout the study, egg numbers and egg weight were recorded daily, laying rate, average daily feed intake (ADFI) and feed conversion ratio (FCR) were calculated weekly. At the end of 1, 6 and 12 weeks, 3 eggs were taken from each replicate to measure egg quality indicators. Eggshell strength was determined using an egg force reader (Orka Food Technology Ltd., Ramat Hasharon, Israel). Eggshell thickness (air cell, equator, and sharp end) was estimated using electronic digital calipers (ABS LUTE 547–360, Mitutoyo, Japan). Haugh unit and albumen height were evaluated using an egg quality analyzer (Orka Food Technology Ltd., Ramat Hasharon, Israel). Yolk color (International commission on illumination L*a*b*, CIELAB) value was assessed using a colorimeter (CR-400, Konica Minolta, Inc., Tokyo, Japan). L*, a*, and b* indicate relative lightness, relative redness, and relative yellowness, respectively.

### Blood and serum biochemical indices

Whole blood samples were immediately transferred to the laboratory of Animal Hospital at China Agricultural University for hematological analysis. Hematology was analyzed using a TEK-II automatic hematology analyzer (Beijing Kangjia Hongyuan Biological Technology Co., Ltd., Beijing, China). Serum lipid metabolism-related indices, including triglyceride (TG), total cholesterol (TC), high-density lipoprotein cholesterol (HDL-C), low-density lipoprotein cholesterol (LDL-C), very low-density lipoprotein cholesterol (VLDL-C), and liver injury biomarkers (AST and ALT), were analyzed according to the manufacturer's instructions (Nanjing Jiancheng Bioengineering Institute, Nanjing, China) using the Mindray BS-370E fully automatic biochemical detection system (Mindray, Shenzhen, China). The activity levels of glutathione peroxidase (GSH-PX), total superoxide dismutase (T-SOD) and total antioxidant oxidase (T-AOC) and malondialdehyde (MDA) were determined using the corresponding kits (Nanjing Jiancheng Bioengineering Institute, Nanjing, China) according to the manufacturer’s instructions.

### Determination of hepatic biochemical indexes and antioxidant capacity

A 0.1 g sample of each liver tissue was homogenized in 0.9 mL phosphate-buffered saline (PBS) with a tissue homogenizer. Then, centrifugation at 4 °C for 10 min was performed, and the supernatant was harvested for biochemical parameter determination. The contents of TG, TC, HDL-C, LDL-C, VLDL-C, and the activity levels of GSH-PX, T-SOD, T-AOC and MDA in supernatant were quantified with the commercial kits (Nanjing Jiancheng Bioengineering Institute, Nanjing, China) following the manufacturer’s protocols. Total protein concentration in the supernatant was determined with Nanjing Jiancheng Total Protein Quantitative Assay Kit (with standard: BCA method) to correct for all liver biochemical parameters.

### Histological observation of liver

The liver tissues were fixed with 4% paraformaldehyde, dehydrated with gradient alcohol and made transparent with xylene. These transparent samples were embedded in paraffin, cut into 5-μm slices, stained with hematoxylin and eosin (H&E), and sheet sealed. The morphology of the liver was observed under a light microscope with different magnification (CK-40, Olympus, Tokyo, Japan).

### Liver Oil Red O staining

For Oil Red O staining, the liver tissues were fixed, embedded in OTC compound and immediately put on the −20 °C quick-freezing machine, sections were cut at 8 μm slide by the CryoStar NX50 HOVPD freezing microtome (Thermo, MA, USA), and stored at −20 °C. The samples were stained with freshly diluted Oil Red O solution for 15 min under dark conditions at room temperature. Following color separation with 60% isopropanol for 20 s and a gentle rinse with water for 3 min, the nuclei were counterstained with hematoxylin for 5 min. After rinsing with distilled water, the slides were sealed with glycerin gelatin. Images of each group were photographed with a light microscope (CK-40, Olympus, Tokyo, Japan).

### RNA sequencing and data analysis

A total of 24 liver samples (6 samples per group) were used for RNA-seq. The primary process is as follows: (1) RNA extraction and quality detection: briefly, total RNA was extracted from liquid nitrogen-frozen liver tissue by using TRIzol (Invitrogen). NanoDrop 2000/N50 detected the purity and concentration of RNA samples, and Agilent 4200 assessed the integrity, to ensure the qualified samples for subsequent transcriptomic sequencing. (2) Library construction and sequencing: Transcriptome sequencing libraries were constructed according to the standard Illumina RNA-seq protocol. After the library was qualified, PE150 mode sequencing was performed using Illumina NovaSeq 6000 sequencing platform (Illumina Inc., San Diego, CA, USA; Berry & Kang Biotechnology Co., Ltd., Beijing, China). (3) Bioinformatic analysis: The raw reads obtained by sequencing were filtered to obtain clean reads, bowtie2 (v.2.3.2) was used to compare clean reads to the silva database to remove rRNA [39]. The remaining reads were compared with the reference genome (Gallus gallus: https://www.ncbi.nlm.nih.gov/datasets/genome/GCF_016699485.2/) using Hisat2 (v.2.2.1) [40]. The ratio of sequencing data to the reference genome was calculated based on the gene location information specified in the genome annotation file (GTF format). FeatureCounts (v.1.6.3) is used to recount the gene levels of each sample respectively [41], and the FPKM value is obtained. (4) Principal component analysis (PCA) represented the relationships and differences between samples by means of linear dimensionality reduction. Differentially expressed genes (DEGs) were identified with edgeR as follows: |log_2_(Fold Change)| > 1.0 and *P-*value < 0.05. These DEGs were enriched by Gene Ontology (GO) and Kyoto Encyclopedia of Genes and Genomes (KEGG) using topGO and KOBAS (v3.0) respectively.

### Proteomic analysis

#### Protein extraction and quality control

Four samples were randomly selected from each group for proteomic analysis. In brief, the steps were as follows: (1) 25 mg of sample was weighed, and transferred into a 2-mL centrifuge tube. Steel ball, lysis solution containing 8 mol/L Urea/50 mmol/L Tris-HCl and Roche cocktail with 1X final concentration were added into the centrifuge tubes, and placed on ice for 5 min. (2) Crush and crack using a grinding instrument (60 Hz, 2 min), centrifuge at 20,000 × *g* and 4 °C for 15 min, and collect the supernatant. (3) Add dithiothreitol (DTT) with a final concentration of 10 mmol/L and bathe in medium water at 37 °C for 1 h. (4) Add iodoacetamide (IAA) with a final concentration of 20 mmol/L and place away from light. (5) Take 10 μg protein solution for each sample and add appropriate loading buffer, mix well, heat at 95 °C for 5 min, centrifuge at 20,000 × *g* for 5 min, take supernatant and add it into the sample hole of 4%–12% SDS polyacrylamide gel. 80 V constant pressure electrophoresis for 20 min, then 120 V constant pressure electrophoresis for 60 min. After the end of electrophoresis, the glue is dyed and decolorized, and it was taken out and photographed, and the quality was judged according to the glue map.

#### Enzymatic digestion

The experimental procedures were as follows: (1) 150 μg protein was taken from each sample. (2) Add 3 μg Trypsin at the ratio of 50:1 (protein:enzyme), enzymolysis at 37 °C for 14–16 h. (3) The enzymolysis peptides were desalted using Waters solid phase extraction column and vacuum-drained. (4) The drained peptides were redissolved in pure water and stored at −20 °C.

#### Quantitative detection (Nano-LC–MS/MS) and analysis

The extracted peptide sample was redissolved with 0.1% FA, centrifuged at 20,000 × *g* for 10 min. The supernatant was collected and injected into a self-loading C18 column (100 μm I.D., 1.8 μm column media particle size, approximately 35 cm column length). Separation was performed by Thermo Scientific EASY-nLC™ 1200 system at a flow rate of 300 nL/min. The liquid phase separated peptides were ionized by nanoESI source and then entered the mass spectrometer Orbitrap Exploris™ 480 (Thermo Fisher Scientific, San Jose, CA, USA). A new data-independent acquisition (DIA) model was adopted for mass spectrometry data acquisition [42]. It has greater advantages than the Data-dependent acquisition (DDA) mode previously adopted by TMT, Label-free and SILAC. The DIA can determine protein molecules with very low abundance in samples more efficiently, greatly improving the reliability of quantitative analysis, and has high quantitative accuracy and repeatability.

DIA-NN software (https://hpc.nih.gov/apps/diann.html) (version 1.8) was used to conduct protein search, identification and quantitative analysis of the DIA mass spectrometry data. The main parameters of DIA-NN are as follows: The protein identification mode was DirectDIA (no actual DDA data was needed to construct the spectral library, and the theoretical virtual spectral library was constructed from the protein sequence library of species Gallus gallus by information analysis method). Then, *t*-test was utilized to identify differentially expressed proteins (DEPs) based on their relative quantitative values (|log_2_(Fold Change)| ≥ 0.263 and *P*-value < 0.05 as the threshold standard). Functional enrichment analysis maps all differential proteins to individual entries annotated in the protein GO and KEGG databases. GSEA (Gene Set Enrichment Analysis) was based on information about the expression levels of all proteins. STRING database (version 11.0) was employed for the analysis of protein–protein interaction (PPI) networks [43].

#### RNA extraction, cDNA synthesis, and qRT-PCR analysis

Total RNA was isolated from liver tissues using TRIzol reagent (Invitrogen). RNA (0.5 µg) was transcribed into cDNA with the Hiscript® III All-in-one RT SuperMix Perfect for qPCR kits (R333-01, Vazyme Biotech Co., Ltd., Nanjing, China). qRT-qPCR was performed using the Applied Biosystems QuantStudio 7 Flex System (Life Technologies, Waltham, USA) following the instructions of Taq Pro Universal SYBR qPCR Master Mix (Q712-02, Vazyme Biotech Co., Ltd., Nanjing, China). mRNA expression levels were normalized to housekeeping genes (*β*-actin and *GAPDH*), and the data were analyzed using the comparative 2^−△△Ct^ method. The primers utilized in the experiment are shown in Table 2.

**Table 2:** Primer sequences for qRT-PCR

Gene	Primer	Primer sequence (5'→3')	Accession No.
*β-Actin*	Forward	TGCGTGACATCAAGGAGAAG	NM_205518
	Reverse	TGCCAGGGTACATTGTGGTA	
*AMPK*	Forward	ATCTGTCTCGCCCTCATCCT	NM_001039603.2
	Reverse	CCACTTCGCTCTTCTTACACCTT	
*ACACA*	Forward	CCGAGAACCCAAAACTACCAG	NM_205505.1
	Reverse	GCCAGCAGTCTGAGCCACTA	
*FASN*	Forward	AAAGCAATTCGTCACGGACA	NM_205155.4
	Reverse	GGCACCATCAGGACTAAGCA	
*PPARA*	Forward	AGGCCAAGTTGAAAGCAGAA	NM_001001464.1
	Reverse	TTTCCCTGCAAGGATGACTC	
*CD36*	Forward	CTGGGAAGGTTACTGCGATT	NM_001030731.1
	Reverse	GCGAGGAACTGTGAAACGATA	
*SREBP1c*	Forward	GCCCTCTGTGCCTTTGTCTTC	NM_204126.2
	Reverse	ACTCAGCCATGATGCTTCTTC	
*SCD1*	Forward	AGCAGAACGAGGCATGGTAG	NM_204890.2
	Reverse	GGATCAGCGTCAGCCCAATA	
*PPARG*	Forward	TGACAGGAAAGACGACAGACA	NM_001001460.2
	Reverse	CTCCACAGAGCGAAACTGAC	
*CPT1A*	Forward	TCGTCTTGCCATGACTGGTG	XM_040700878.2
	Reverse	GCTGTGGTGTCTGACTCGTT	
*HMGCR*	Forward	CATAGGTGGCTACAACG	NM_204485.3
	Reverse	TACGCTCCATCAAAGTG	
*ChREBP*	Forward	GATGAGCACCGCAAACCAGAGG	NM_001398177.1
	Reverse	TCGGAGCCGCTTCTTGTAGTAGG	
*LXRα*	Forward	CAAAGGGAATGAATGAGC	NM_204542.3
	Reverse	AGCCGAAGGGCAAACAC	
*FXR*	Forward	AGTAGAAGCCATGTTCCTCCGTT	NM_001396910.1
	Reverse	GCAGTGCATATTCCTCCTGTGTC	
*ACOX1*	Forward	ATGTCACGTTCACCCCATCC	NM_001006205.2
	Reverse	AGGTAGGAGACCATGCCAGT	
*SIRT1*	Forward	TAGCCAATGGTTTCCACTCC	NM_001004767.2
	Reverse	AAGAATTGTCCGTGGGTCTG	
*FABP3*	Forward	TCTGAAAGCTCTTGCACTGCC	NM_001030889.2
	Reverse	TCACAGTCTGCCTGGGTGTT	
*FABP4*	Forward	GCACCTGGAAGCTCCTTTCT	NM_204290.2
	Reverse	ATTAGGCTTGGCCACACCAG	
*SCP2*	Forward	AGGAGGCAACCTGGGTAGT	NM_001305200.2
	Reverse	ATTTGCCTTGAAAGAAGGCTGTC	
*ABCA1*	Forward	TCAATCACCCGCTCAACT	NM_204145.3
	Reverse	CTGGCAGGAACAAAGGAC	

### Statistical analysis

SPSS 27.0 software (SPSS Inc., Chicago, IL, USA) was utilized to conduct one-way ANOVA and Duncan multiple comparison of all data, encompassing laying hen performance, egg quality, biochemical indexes, antioxidant and anti-inflammatory indices, etc. Results were presented as mean ± SEM (standard error of the mean); *n* = 10 hens per group. GraphPad Prism 9.5 (GraphPad Software, San Diego, CA, USA) was employed for data visualization. Statistical significance was considered at a *P*-value threshold less than 0.05.

## Results

### CA and CGA enhanced laying performance in laying hens

Laying performance was recorded daily during the period of test. As illustrated in Table 3, the supplementation groups of CA and CGA exhibited noticeably higher egg laying rate compared to the CON group from week 1 to 6. Moreover, compared to the CON group, the average egg weight was significantly increased in the CA25, CGA200 and CGA400 groups. The data also showed that one-eighth of the CA additive dose achieved an equivalent improvement as CGA by comparing the CA25 group with the CGA200 group in terms of egg-laying rate and average egg weight. However, ADFI and FCR were not strikingly altered. However, these differences were only observed in the first 6 weeks, and sustained better performance was not observed in all CA and CGA groups from week 7 to 12. A dose dependent effect of CA was also not found. In summary, dietary CA and CGA contributed to enhanced egg-laying performance.

**Table 3:** Effect of the dietary CA and CGA on the laying performance

Item	CON	CA25	CA50	CA100	CA200	*P*-value	CON	CGA100	CGA200	CGA400	*P*-value
Weeks 1–6											
Laying rate, %	92.23 ± 0.33^c^	93.27 ± 0.26^a^	92.56 ± 0.15^bc^	92.22 ± 0.27^c^	92.76 ± 0.28^ab^	0.002	92.23 ± 0.33^b^	92.97 ± 0.37^a^	93.32 ± 0.34^a^	93.06 ± 0.44^a^	0.011
Average egg weight, g	64.35 ± 0.35^b^	65.08 ± 0.17^a^	64.32 ± 0.06^b^	64.44 ± 0.07^ab^	64.27 ± 0.19^b^	0.052	64.35 ± 0.35^b^	64.24 ± 0.32^b^	65.11 ± 0.20^a^	65.71 ± 0.16^a^	0.002
ADFI, g/hen/d	131.46 ± 2.47	131.53 ± 2.73	129.34 ± 2.68	128.35 ± 3.10	130.84 ± 3.00	0.861	131.46 ± 2.47	130.55 ± 3.10	128.83 ± 4.16	129.99 ± 2.63	0.910
FCR, g/g	2.22 ± 0.03	2.17 ± 0.05	2.18 ± 0.05	2.16 ± 0.06	2.20 ± 0.05	0.877	2.22 ± 0.03	2.19 ± 0.04	2.12 ± 0.07	2.13 ± 0.05	0.303
Weeks 7–12											
Laying rate, %	89.21 ± 0.59	90.12 ± 0.65	89.02 ± 0.75	89.87 ± 0.75	89.55 ± 0.80	0.662	89.21 ± 0.59	89.77 ± 0.62	90.15 ± 0.69	90.00 ± 0.94	0.714
Average egg weight, g	65.76 ± 0.29^a^	65.80 ± 0.06^a^	65.79 ± 0.24^a^	64.99 ± 0.15^b^	65.26 ± 0.08^b^	0.004	65.76 ± 0.29	65.48 ± 0.17	65.60 ± 0.15	65.81 ± 0.07	0.414
ADFI, g/hen/d	135.53 ± 0.79	136.26 ± 1.14	134.64 ± 1.15	135.59 ± 1.48	133.45 ± 1.04	0.46	135.53 ± 0.79	134.44 ± 2.00	136.87 ± 1.28	137.45 ± 1.84	0.492
FCR, g/g	2.31 ± 0.02	2.30 ± 0.03	2.31 ± 0.02	2.31 ± 0.04	2.29 ± 0.03	0.929	2.31 ± 0.02	2.29 ± 0.04	2.32 ± 0.03	2.32 ± 0.05	0.910

The assessment measures were conducted at weeks 1–12. All data are presented as mean ± SEM (*n* = 10)

^a−c^Means with different superscripts within the groups differ significantly (*P* < 0.05)

### CA and CGA improved egg quality in laying hens

The results of egg quality determination are presented in Table 4. At the end of weeks 1, 6, and 12, the albumen height and Haugh unit were enhanced in CA and CGA groups compared with the CON group, but shell strength, shell thickness, and yolk color were not significantly altered. However, yolk lightness (L*), yolk redness (a*), and yolk yellowness (b*) were reduced in CA and CGA groups, compared to the CON group. The results also indicated that a quarter of the added CA had an equivalent effect to CGA (comparing CA25 and CGA100) on albumen height and Haugh unit from week 1 to week 6. Overall, dietary CA and CGA resulted in promoted egg quality compared to CON group.

**Table 4:** Effect of the dietary CA and CGA on the egg quality

Item	CON	CA25	CA50	CA100	CA200	*P*-value	CON	CQA100	CQA200	CQA400	*P*-value
Week 1											
Shell strength, kg/m^2^	3.55 ± 0.12	3.43 ± 0.21	3.54 ± 0.14	3.50 ± 0.33	3.59 ± 0.18	0.563	3.55 ± 0.12	3.48 ± 0.45	3.50 ± 0.22	3.49 ± 0.39	0.421
Shell thickness, mm	0.45 ± 0.02	0.45 ± 0.01	0.52 ± 0.01	0.50 ± 0.01	0.44 ± 0.01	0.689	0.45 ± 0.02	0.43 ± 0.01	0.45 ± 0.01	0.42 ± 0.01	0.356
Albumen height, mm	5.61 ± 0.25^b^	6.30 ± 0.20^a^	6.34 ± 0.32^a^	6.46 ± 0.17^a^	6.47 ± 0.23^a^	0.006	5.61 ± 0.25^c^	6.87 ± 0.15^a^	6.38 ± 0.15^b^	6.12 ± 0.25^b^	< 0.001
Haugh unit	73.46 ± 2.08^b^	77.11 ± 1.67^a^	77.46 ± 2.85^a^	78.75 ± 1.49^a^	78.44 ± 1.73^a^	0.021	73.46 ± 2.08^b^	80.64 ± 1.07^a^	79.54 ± 1.38^a^	75.77 ± 2.80^b^	< 0.001
Yolk color	6.07 ± 0.31	5.87 ± 0.27	5.70 ± 0.29	5.87 ± 0.31	5.43 ± 0.26	0.545	6.07 ± 0.31	5.73 ± 0.28	5.93 ± 0.30	6.23 ± 0.29	0.647
L*	64.52 ± 0.51	64.11 ± 0.47	63.61 ± 0.48	63.79 ± 0.42	63.92 ± 0.76	0.777	64.52 ± 0.51	63.71 ± 0.47	63.58 ± 0.51	63.48 ± 0.46	0.393
a*	2.84 ± 0.21^a^	2.46 ± 0.17^b^	2.24 ± 0.19^b^	2.33 ± 0.19^b^	2.29 ± 0.19^b^	0.042	2.84 ± 0.21^a^	2.01 ± 0.15^c^	2.38 ± 0.14^bc^	2.71 ± 0.16^ab^	< 0.001
b*	47.13 ± 0.55^a^	46.05 ± 0.68^ab^	43.60 ± 0.72^c^	44.58 ± 0.77^bc^	44.33 ± 0.90^bc^	0.003	47.13 ± 0.55^a^	43.75 ± 0.72^c^	44.36 ± 0.89^bc^	45.67 ± 0.54^ab^	0.003
Week 6											
Shell strength, kg/m^2^	3.52 ± 0.23	3.43 ± 0.15	3.52 ± 0.12	3.49 ± 0.13	3.56 ± 0.24	0.429	3.52 ± 0.23	3.47 ± 0.52	3.50 ± 0.13	3.50 ± 0.39	0.663
Shell thickness, mm	0.43 ± 0.01	0.44 ± 0.02	0.43 ± 0.01	0.43 ± 0.01	0.45 ± 0.02	0.567	0.43 ± 0.01	0.45 ± 0.01	0.42 ± 0.01	0.48 ± 0.01	0.456
Albumen height, mm	5.33 ± 0.40^b^	6.39 ± 0.25^a^	6.17 ± 0.35^a^	6.21 ± 0.24^a^	5.82 ± 0.21^ab^	0.028	5.33 ± 0.40^b^	6.25 ± 0.27^a^	6.25 ± 0.29^a^	5.80 ± 0.25^b^	0.029
Haugh unit	73.39 ± 3.63^b^	77.12 ± 2.30^ab^	77.27 ± 2.21^ab^	79.04 ± 1.96^a^	74.78 ± 2.24^b^	0.033	73.39 ± 3.63	75.43 ± 2.69	75.75 ± 2.54	74.16 ± 2.00	0.672
Yolk color	6.00 ± 0.34^a^	4.58 ± 0.32^b^	4.17 ± 0.14^b^	4.13 ± 0.12^b^	4.42 ± 0.20^b^	< 0.001	6.00 ± 0.34^a^	4.29 ± 0.11^b^	4.38 ± 0.12^b^	4.54 ± 0.11^b^	< 0.001
L*	79.08 ± 0.49^a^	79.47 ± 0.42^a^	80.16 ± 0.31^a^	79.38 ± 0.59^a^	75.44 ± 0.56^b^	< 0.001	78.71 ± 0.55	79.15. ± 0.46	77.83 ± 0.55	78.27 ± 0.34	0.208
a*	5.34 ± 0.75^a^	4.04 ± 0.36^b^	3.89 ± 0.40^b^	3.91 ± 0.26^b^	3.50 ± 0.27^b^	< 0.001	5.34 ± 0.75^a^	3.57 ± 0.24^b^	3.49 ± 0.26^b^	3.95 ± 0.26^b^	< 0.001
b*	62.69 ± 1.18^a^	63.15 ± 1.07^a^	63.95 ± 0.95^a^	62.43 ± 1.33^a^	56.42 ± 0.89^b^	< 0.001	62.69 ± 1.18	59.59 ± 0.65	60.09 ± 0.84	60.19 ± 0.70	0.060
Week 12											
Shell strength, kg/m^2^	3.52 ± 0.26	3.40 ± 0.19	3.51 ± 0.19	3.49 ± 0.25	3.52 ± 0.14	0.657	3.52 ± 0.26	3.47 ± 0.43	3.49 ± 0.18	3.48 ± 0.61	0.684
Shell thickness, mm	0.43 ± 0.02	0.42 ± 0.01	0.44 ± 0.01	0.41 ± 0.02	0.46 ± 0.02	0.567	0.43 ± 0.02	0.43 ± 0.02	0.44 ± 0.01	0.48 ± 0.02	0.775
Albumen height, mm	5.88 ± 0.23^b^	6.54 ± 0.23^a^	6.04 ± 0.28^ab^	6.23 ± 0.28^ab^	6.24 ± 0.33^ab^	0.206	5.88 ± 0.23	6.07 ± 0.29	6.26 ± 0.19	6.31 ± 0.34	0.406
Haugh unit	74.13 ± 1.80	77.25 ± 1.58	75.75 ± 2.80	76.06 ± 2.31	77.03 ± 2.85	0.508	74.13 ± 1.80	76.13 ± 2.68	77.15 ± 1.40	77.24 ± 2.72	0.350
Yolk color	6.08 ± 0.38	6.13 ± 0.29	6.21 ± 0.24	5.63 ± 0.34	5.50 ± 0.26	0.222	6.08 ± 0.38	5.67 ± 0.24	5.71 ± 0.20	5.79 ± 0.31	0.507
L*	65.67 ± 0.57	66.63 ± 0.40	64.77 ± 0.54	65.59 ± 0.69	65.99 ± 0.60	0.277	65.67 ± 0.57^bc^	67.76 ± 0.41^a^	66.13 ± 0.61^ab^	64.16 ± 0.52^c^	< 0.001
a*	2.10 ± 0.48^a^	1.64 ± 0.18^ab^	1.64 ± 0.19^ab^	1.22 ± 0.14^ab^	1.33 ± 0.21^b^	0.018	2.10 ± 0.48	1.66 ± 0.22	1.21 ± 0.16	1.34 ± 0.24	0.140
b*	50.89 ± 1.26	51.46 ± 1.43	48.31 ± 1.01	49.47 ± 1.08	51.29 ± 1.12	0.217	50.89 ± 1.26^bc^	54.82 ± 0.70^a^	52.61 ± 0.89^ab^	48.23 ± 1.15^c^	< 0.001

L* = lightness; a* = redness; b* = yellowness. The assessment measures were conducted at weeks 1, 6 and 12. All data are presented as mean ± SEM (*n* = 10)

^a−c^Means with different superscripts within the groups differ significantly (*P* < 0.05)

### CA and CGA changed hematology indices

Table 5 presents the hematology indices at the end of week 12. Generally speaking, hematological indicators are vital for assessing laying hens’ health, revealing immune function, hematopoiesis, and stress response. Increases in white blood cells (WBC) indicate infections or immune responses, while changes in red blood cells (RBC) suggest anemia from deficiencies or health issues. Here, our results shown that the addition of CA and CGA significantly decreased the WBC compared to the CON group, yet it did not alter the RBC, indicating that CA and CGA benefited the immunity of laying hens with basically no health issues. The other blood parameters were not significantly influenced by CA25 compared to the CON group. Additionally, excluding middle cell absolute value (MID#), hemoglobin (HGB), mean corpuscular hemoglobin (MCH), mean corpuscular hemoglobin (MCHC) and platelet distribution width (PDW), CGA also had no significant effect on blood when its addition did not exceed 200 mg/kg compared to the CON group. In summary, addition of CA and CGA has positive significance in improving the immunity of laying hens.

**Table 5:** Effect of the dietary CA and CGA on the routine blood test

Item	CON	CA25	CA50	CA100	CA200	*P*-value	CON	CGA100	CGA200	CGA400	*P*-value
WBC	117.75 ± 0.50^a^	114.02 ± 0.86^b^	113.36 ± 1.20^b^	116.39 ± 0.4^ab^	113.61 ± 1.65^b^	0.004	117.75 ± 0.50^a^	114.16 ± 0.96^b^	113.78 ± 0.826^b^	111.76 ± 1.026^b^	< 0.001
LYM#	46.78 ± 1.51^b^	49.05 ± 0.81^ab^	51.64 ± 1.11^a^	46.37 ± 0.65^b^	48.27 ± 1.22^b^	< 0.001	46.78 ± 1.51	47.50 ± 0.85	47.55 ± 1.12	48.43 ± 1.20	0.654
MID#	16.88 ± 0.61^ab^	16.85 ± 0.11^ab^	16.99 ± 0.11^a^	16.64 ± 0.14^ab^	16.52 ± 0.17^b^	0.048	16.88 ± 0.61^a^	16.53 ± 0.08^b^	16.58 ± 0.09^b^	16.41 ± 0.06^b^	< 0.001
GRA#	54.09 ± 0.91^a^	48.11 ± 1.56^ab^	44.73 ± 2.21^b^	53.38 ± 1.01^a^	48.83 ± 2.67^ab^	< 0.001	54.09 ± 0.91^a^	50.13 ± 1.7^ab^	49.65 ± 1.98^ab^	46.92 ± 2.20^b^	0.039
LYM%	39.83 ± 0.54^b^	43.20 ± 0.98^ab^	45.78 ± 1.39^a^	39.94 ± 0.63^b^	42.77 ± 1.65^ab^	< 0.001	39.83 ± 0.54	41.80 ± 1.10	41.98 ± 1.30	43.58 ± 1.48	0.140
MID%	14.29 ± 0.08^b^	14.73 ± 0.13^ab^	14.94 ± 0.14^a^	14.27 ± 0.15^b^	14.50 ± 0.17^ab^	0.002	14.29 ± 0.08	14.43 ± 0.12	14.53 ± 0.17	14.64 ± 0.15	0.265
GRA%	45.88 ± 0.62^a^	42.07 ± 1.10^ab^	39.28 ± 1.52^b^	45.79 ± 0.76^a^	42.73 ± 1.79^ab^	< 0.001	45.88 ± 0.62	43.77 ± 1.20	43.48 ± 1.45	41.78 ± 1.62	0.146
RBC	3.00 ± 0.06	2.97 ± 0.08	3.00 ± 0.11	2.96 ± 0.05	3.10 ± 0.08	0.669	3.00 ± 0.06	2.90 ± 0.08	3.03 ± 0.07	2.97 ± 0.09	0.659
HGB	116.25 ± 2.07	114.67 ± 3.82	116.90 ± 4.19	114.45 ± 2.51	121.60 ± 2.40	0.468	116.25 ± 2.07^a^	113.17 ± 3.45^a^	63.75 ± 6.69^b^	50.17 ± 2.81^b^	< 0.001
HCT	0.28 ± 0.01	0.28 ± 0.01	0.28 ± 0.01	0.27 ± 0.00	0.29 ± 0.01	0.597	0.28 ± 0.01	0.27 ± 0.01	0.29 ± 0.01	0.28 ± 0.01	0.319
MCV	92.98 ± 0.63	94.67 ± 1.18	92.36 ± 0.91	93.02 ± 0.9	93.02 ± 1.16	0.474	92.98 ± 0.63	94.81 ± 0.92	95.89 ± 1.04	93.18 ± 1.10	0.084
MCH	38.71 ± 0.29	38.54 ± 0.58	38.87 ± 0.54	38.59 ± 0.40	39.61 ± 0.48	0.499	38.71 ± 0.29^a^	38.83 ± 0.38^a^	21.33 ± 2.66^b^	16.69 ± 0.57^b^	< 0.001
MCHC	395.33 ± 3.01	386.83 ± 6.13	399.50 ± 4.15	394.09 ± 4.07	400.10 ± 4.60	0.208	395.33 ± 3.01^a^	389.25 ± 5.56^a^	211.17 ± 26.16^b^	170.50 ± 6.28^b^	< 0.001
RDW-SD	17.10 ± 3.13	24.98 ± 4.41	20.85 ± 3.96	21.21 ± 3.74	28.25 ± 3.75	0.261	17.10 ± 3.13	27.77 ± 3.40	25.22 ± 3.48	23.80 ± 3.42	0.128
RDW-CV	29.58 ± 0.58	28.99 ± 0.50	29.17 ± 0.93	29.29 ± 0.88	28.86 ± 0.89	0.961	29.58 ± 0.58	29.58 ± 0.56	30.64 ± 0.97	30.28 ± 0.56	0.592
PLT	21.00 ± 2.46^b^	31.18 ± 2.49^ab^	28.56 ± 5.88^b^	27.18 ± 2.62^b^	44.75 ± 6.67^a^	0.002	21.00 ± 2.46	28.00 ± 3.02	31.64 ± 3.46	33.36 ± 6.76	0.179
PCT	0.02 ± 0.00	0.03 ± 0.00	0.05 ± 0.02	0.03 ± 0.00	0.04 ± 0.01	0.184	0.02 ± 0.00	0.03 ± 0.00	0.04 ± 0.01	0.03 ± 0.01	0.089
MPV	0.97 ± 0.06	11.00 ± 0.09	10.68 ± 0.19	10.87 ± 0.07	10.88 ± 0.11	0.237	10.97 ± 0.06^ab^	10.91 ± 0.07^ab^	11.14 ± 0.07^a^	10.78 ± 0.13^b^	0.036
PDW	77.75 ± 5.15^a^	63.38 ± 3.57^abc^	59.03 ± 6.28^bc^	69.19 ± 5.16^ab^	50.66 ± 3.89^c^	0.002	77.75 ± 5.15^a^	65.73 ± 4.86^ab^	55.31 ± 6.17^b^	72.41 ± 5.42^ab^	0.024

*WBC * White blood cells count, *LYM#* Lymphocyte absolute value, *MID#* Middle cell absolute value, *GRA#* Granulocyte absolute value, *LYM%* Lymphocyte percentage, *MID%* Middle cell percentage, *GRA%* Granulocyte percentage, *RBC* Red blood cells count, *HGB* Hemoglobin, *HCT* Hematocrit, *MCV* Mean corpuscular volume, *MCH* Mean corpuscular hemoglobin, *MCHC* Mean corpuscular hemoglobin concentration, *RDW-SD* Red cell distribution width of standard deviation, *RDW-CV* Red cell distribution width coefficient of variation, *PLT* Platelets, *PCT* Platelet crit, *MPV* Mean platelet volume, *PDW* Platelet distribution width

The assessment measures were conducted at week 12. All data are presented as mean ± SEM (*n *= 10)

^a−c^Means with different superscripts within the groups differ significantly (*P* < 0.05)

### CA and CGA ameliorated the levels of serum lipid metabolism and antioxidant activity

**Fig. 1:**
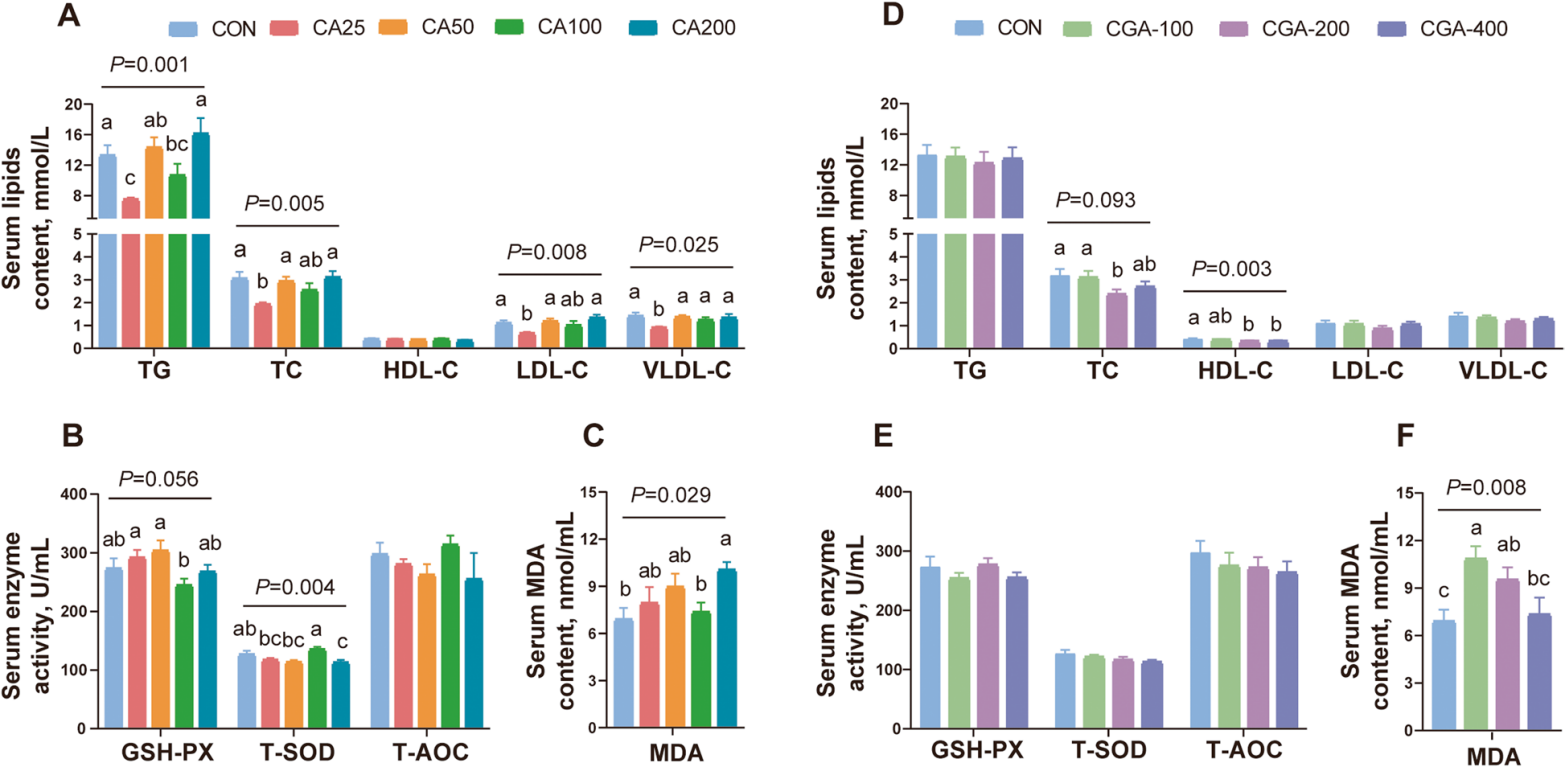
Effects of CA and CGA on serum lipid metabolism levels and antioxidant activity. **A** and **D** Serum TG, TC, HDL-C, LDL-C and VLDL-C levels (*n* = 10). **B** and **E** Serum GSH-PX, T-SOD and T-AOC activity levels (*n* = 10). **C** and **F** Serum MDA content (*n* = 10). Data are presented as the mean ± SEM. ^a−c^Means with different letter differ significantly (*P* < 0.05)

The serum lipid metabolism and antioxidant activity were assessed at the end of week 12. As presented in Fig. 1A, compared with the CON group, serum TG, TC, LDL-C, and VLDL-C levels were significantly reduced in the CA25 group, but no significant differences were observed at higher concentrations of CA, including serum HDL-C levels. However, compared to the CON group, serum TG, LDL-C, and VLDL-C levels were not significantly affected by CGA groups except for a significant reduction in serum TC and an increase in HDL-C in the CGA200 and CGA400 groups (Fig. 1D). However, compared to the CON group, the serum antioxidant system (GSH-PX, T-SOD and T-AOC) was not altered, but dietary CA and CGA resulted in an increase in the level of the oxidant cue (MDA) in most of CA and CGA groups (Fig. 1B, C, E, and F). The results also established that the CA25 group had the best effect on reducing serum lipid levels compared with other groups.

### CA and CGA alleviated hepatic lipid accumulation and liver damage

**Fig. 2:**
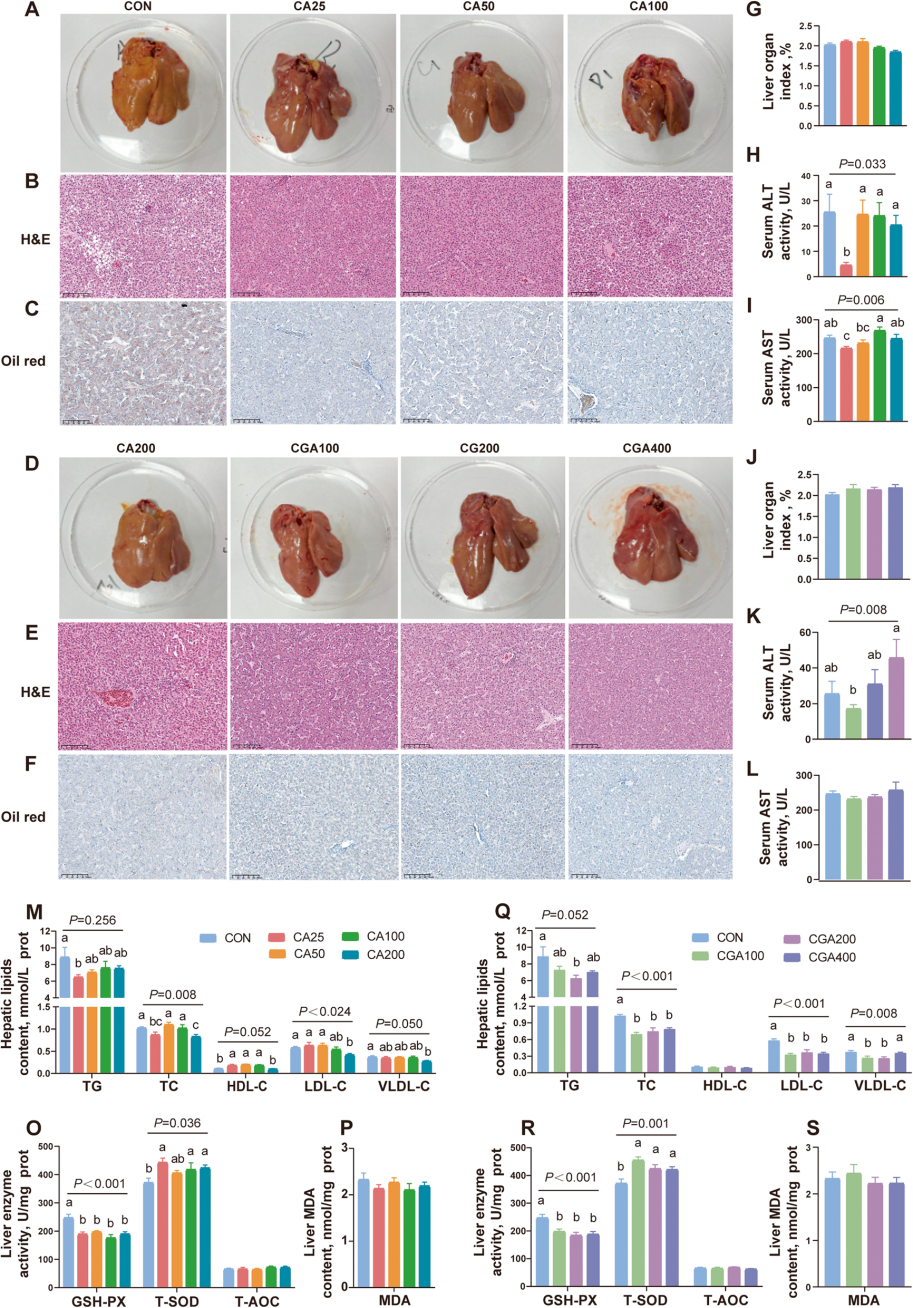
CA and CGA alleviated hepatic lipid accumulation and liver damage. **A **and** D** liver morphology (*n* = 6). **B **and** E** liver H&E staining (*n* = 6). **C **and** F** Hepatic Oil Red O staining (*n* = 6). **G **and** J** (Liver index = liver weight/body weight) × 100 (*n* = 10). **H** and **K** Serum ALT activity level (*n* = 10). **I **and** L** Serum AST activity level (*n* = 10). **M **and** Q** Liver TG, TC, HDL-C, LDL-C and VLDL-C levels (*n* = 10). **O** and **R** Hepatic GSH-PX, T-SOD and T-AOC activity levels (*n* = 10). **P **and **S** Liver MDA content (*n* = 10). ^a−c^Means with different letters differ significantly (*P* < 0.05)

To evaluate the therapeutic effects of CA and CGA on fatty liver, focused analysis was conducted using liver specimens. Liver tissue H&E staining and phenotype images revealed that the CON group exhibited hepatic steatosis with yellowish liver and numerous fat vacuoles. However, this deterioration was significantly attenuated by administration of CA and CGA (Fig. 2A, B, D, and E). Meanwhile, CA and CGA had no significant effect on liver index compared with the CON group (Fig. 2G and J). Oil Red O staining indicated that the liver tissues in the CA and CGA groups had lower lipid accumulation compared to the CON group (Fig. 2C and F). ALT and AST activities, which are serum indicators used to assess liver damage [44], were significantly decreased in the CA25 and CGA100 groups compared to the CON group, suggesting alleviation of liver damage (Fig. 2H, I, K, and L). Additionally, intrahepatic TG, TC, LDL-C contents were decreased, while HDL-C content increased in the CA and CGA groups compared to the CON group. These data suggest that CA and CGA have regulatory effects on hepatic lipid metabolism (Fig. 2M and Q). The effects of CA and CGA on the liver antioxidant system are presented in Fig. 2O, P, R, and S. Compared to the CON group, T-SOD antioxidant activity was significantly enhanced in the CA and CGA groups, but dietary CA and CGA resulted in a remarkable decrease in GSH-PX activity. In summary, as established by the above results, dietary CA and CGA can effectively mitigate liver injury by inhibiting fat deposition and enhancing some antioxidant capacities. Furthermore, comparing the CA25 group with the CGA200 group, particularly regarding TG and TC contents, the results revealed that one-eighth the dosage of CA could achieve significant effects comparable to CGA.

### CA and CGA regulated hepatic gene expression profiles

**Fig. 3:**
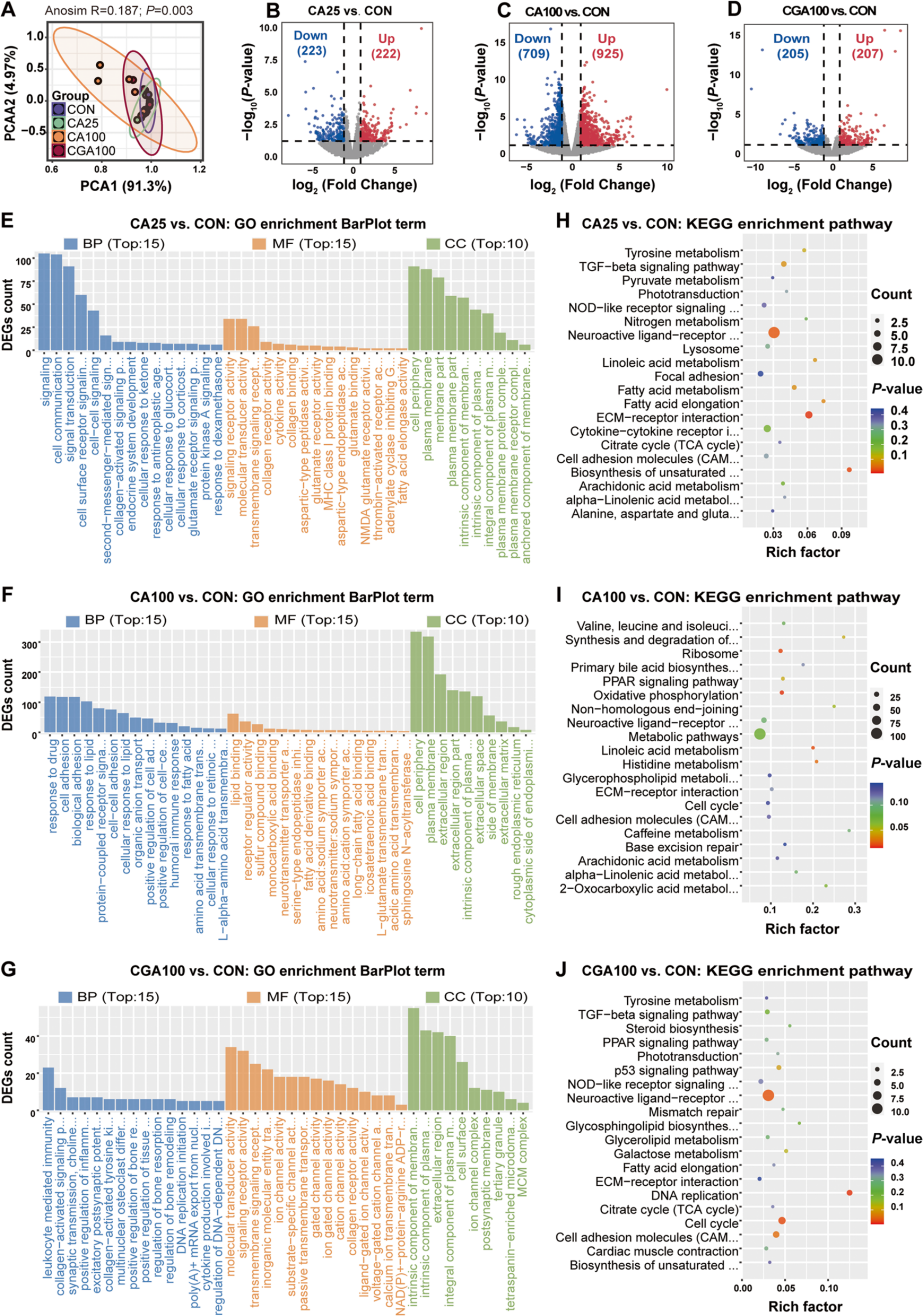
CA and CGA regulated hepatic gene expression profiles. **A** PCA analysis. **B–D** Volcano plot of DGEs (*n* = 6). **E–G** GO term enrichment analysis of DEGs. BP: Biological Process, MF: Molecular Function, CC: Cellular Component (*n* = 6). **H–J** KEGG pathway enrichment analysis of DEGs. *n* = 6 hens per group

To elucidate the underlying mechanisms by which CA and CGA mitigate fatty liver, we examined their regulatory impact on hepatic gene transcription. Notably, our integrated analysis revealed that superior therapeutic effects were observed in both the CA25 and CGA100 groups. Additionally, to conduct a more nuanced comparison at the same dosage, the CA100 group was also subjected to transcriptomic analysis. Statistics of transcriptome sequencing were indicated that the data reliable for further analysis (Table S1). As depicted in Fig. 3A, PCA demonstrated that the gene expression profiles of the CON, CA25, CA100, and CGA100 groups were largely analogous. However, in group-wise comparison, CA25, CA100, and CGA100 manifested 445, 1,634, and 412 differentially expressed genes (DEGs), respectively compared to the CON (Fig. 3B–D, Tables S2–S4). The DEGs were utilized for GO terms enrichment analysis, including Biological Process (BP), Molecular Function (MF), and Cellular Component (CC). The top 10–15 GO terms jointly enriched in CA25 and CGA100 groups were collagen-activated signaling pathway, signaling receptor activity, collagen receptor activity, cell periphery and intrinsic component of membrane (Fig. 3E–G, Tables S5–S7). Moreover, KEGG pathway analysis indicated that among the top 20 pathways, there were several pathways collectively enriched in CA25 and CGA100 groups, including biosynthesis of unsaturated fatty acids, citrate cycle (TCA cycle), fatty acid elongation, fatty acid metabolism, cell adhesion molecules (CAMs), ECM-receptor interaction, neuroactive ligand-receptor interaction, NOD-like receptor signaling pathway, and TGF-beta signaling pathway, predominantly focusing on fatty acid metabolism and immune responses (Fig. 3H, J, Tables S8, S10). Similarly, some of the same enriched pathways also existed in the CA100 group (Fig. 3I, Table S9). These results suggested that CA and CGA may operate under similar underlying mechanisms to alleviate fatty liver in laying hens.

### CA and CGA modulated hepatic protein expression profiles

**Fig. 4:**
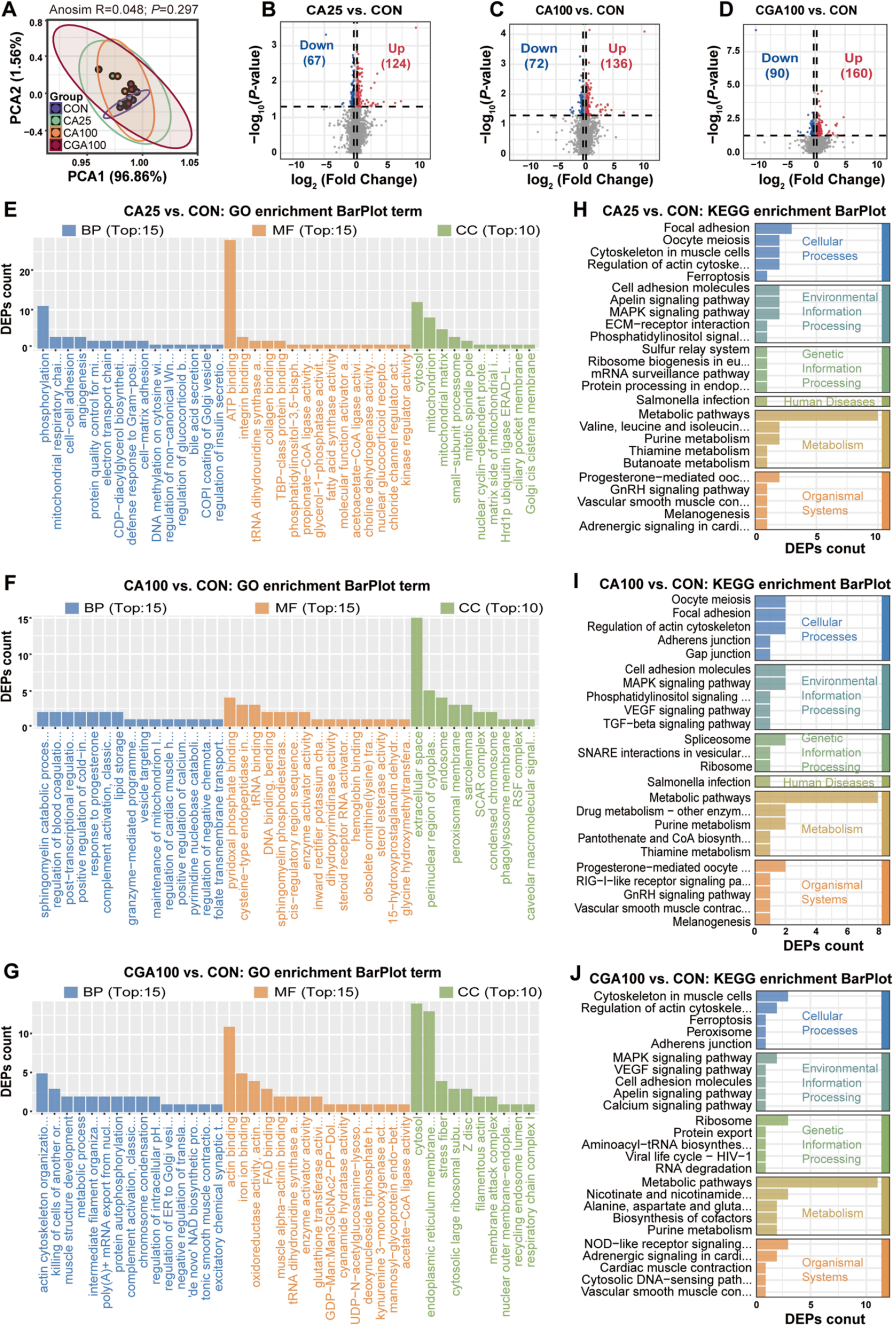
CA and CGA regulated hepatic protein expression profiles. **A** PCA analysis. **B**–**D** Volcano plot of DGPs (*n* = 6). **E–G** GO term enrichment analysis of DEPs. BP: Biological Process, MF: Molecular Function, CC: Cellular Component (*n* = 6). **H**–**J** KEGG pathway enrichment analysis of DEPs. *n* = 4 hens per group

To further elucidate the mechanisms underlying the alleviation of fatty liver by CA and CGA, proteomic analyses were conducted on CON, CA25, CA100, and CGA100 groups. As illustrated in Fig. 4A, PCA analysis revealed similar protein expression profiles across these groups. However, in group-wise analysis, CA25, CA100, and CGA100 exhibited 191, 208, and 250 differentially expressed proteins (DEPs), respectively, compared to the CON group (Fig. 4B–D, Tables S11–S13). GO term enrichment analysis mainly belonging to Biological Process (BP), Molecular Function (MF), and Cellular Component (CC). Among them in the CA25 group, the significant top 10–15 terms including phosphorylation, mitochondrial respiratory chain complex I assembly, electron transport chain, CDP-diacylglycerol biosynthetic process, bile acid secretion, regulation of insulin secretion involved in cellular response to glucose stimulus, ATP binding, phosphatidylinositol-3,5-bisphosphate phosphatase activity, propionate-CoA ligase activity, glycerol-1-phosphatase activity, fatty acid synthase activity, acetoacetate-CoA ligase activity, mitochondrion, and mitochondrial matrix, which are closely related to lipid synthesis and lipid oxidation (Fig. 4E, Table S14). Moreover, the significant top 10–15 terms of relating to lipid synthesis and lipid oxidation, including metabolic process, protein autophosphorylation, oxidoreductase activity, enzyme activator activity, and respiratory chain complex I, were enriched in the CGA100 group (Fig. 4G, Table S16). These related significant terms, including sphingomyelin catabolic process, lipid storage, maintenance of mitochondrion location, sphingomyelin phosphodiesterase, steroid receptor RNA activator RNA binding activity, sterol esterase activity, and peroxisomal membrane also illustrated in the CA100 group (Fig. 4F, Table S15). KEGG analysis indicated that DEPs in the CA25, CA100, and CGA100 groups were enriched in 51, 44, and 45 pathways (Tables S17–S19), respectively. Furthermore, these top 5 pathways were highly similar, among which ferroptosis, cell adhesion molecules, apelin signaling pathway, MAPK signaling pathway, and metabolic pathways were closely related to lipid metabolism and immunity (Fig. 4H–J, Tables S17–S19).

**Fig. 5:**
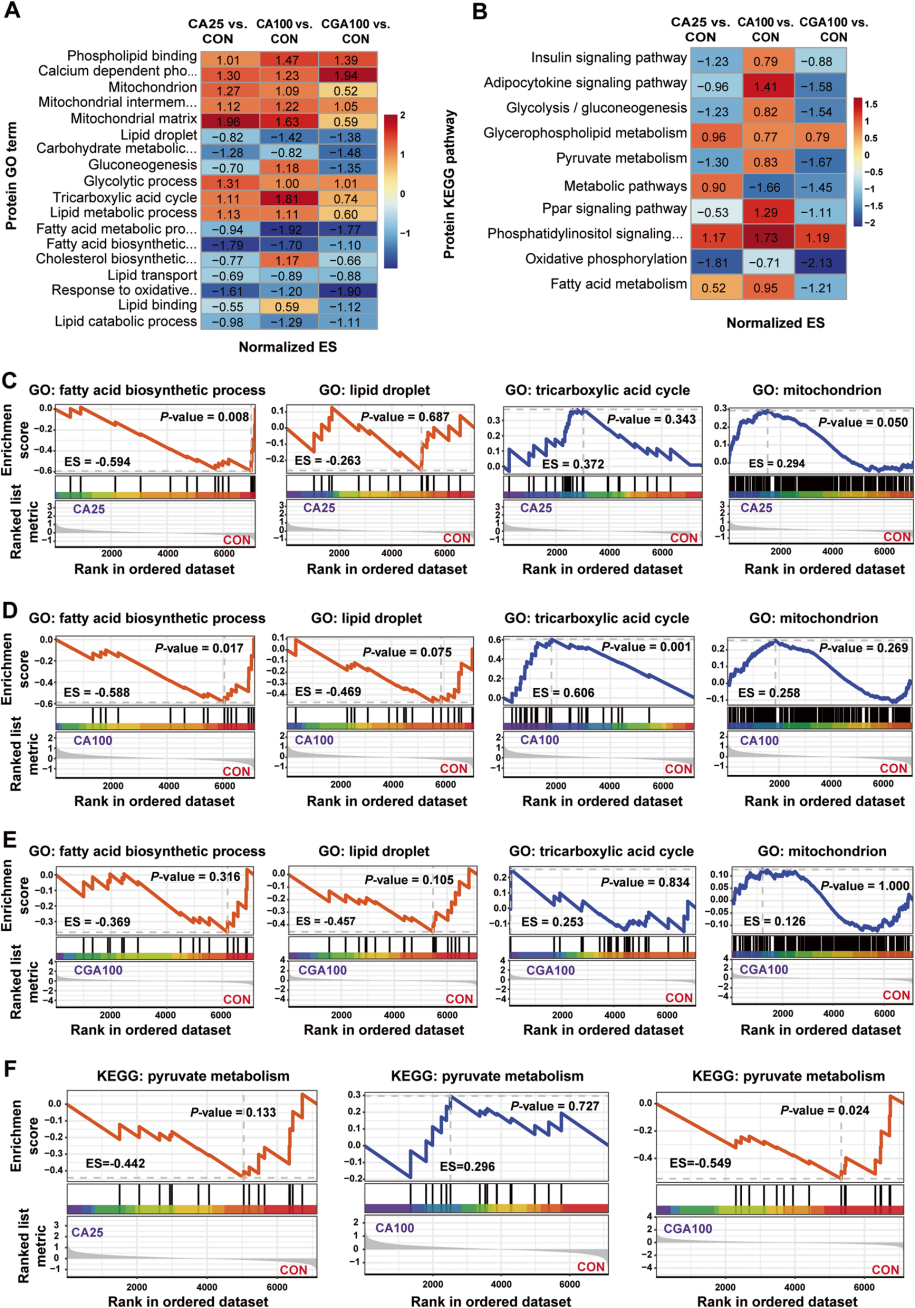
GO term and KEGG pathway of GSEA (Gene Set Enrichment Analysis) enrichment analysis using all proteins. **A** GO term of GSEA enrichment analysis related to lipid metabolism.** B** KEGG pathway of GSEA enrichment analysis related to lipid metabolism. **C–E** GO term of GSEA enrichment analysis in fatty acid biosynthetic, lipid droplet, tricarboxylic acid cycle and mitochondrion. **F** KEGG pathway of GSEA enrichment analysis in pyruvate metabolism. A positive ES (enrichment score) value means that the term or pathway is up-regulated, whereas a negative ES value means that term or pathway is down-regulated. *n* = 4 hens per group

For comprehensive analysis, all expressed proteins, beyond merely DEPs, underwent GSEA enrichment analysis. Figure 5A revealed that GO terms enriched in these proteins showed downregulation of lipid synthesis pathways (including lipid droplet, carbohydrate metabolic process, fatty acid metabolic process, fatty acid biosynthetic process, cholesterol biosynthetic process, and lipid binding) and upregulation of lipid oxidation metabolism pathways (including mitochondrion, mitochondrial intermembrane space, mitochondrial matrix, tricarboxylic acid cycle, lipid metabolic process) (Table S20). This trend was consistently mirrored in KEGG pathways through GSEA enrichment analysis (Fig. 5B, Table S21). Specifically, some of the pathways, such as fatty acid biosynthetic process, lipid droplet formation, tricarboxylic acid cycle, and mitochondrial function, were highlighted in Fig. 5C–F (Tables S20 and S21).

### CA and CGA reduced hepatic lipid deposition by activating the ADPN-AMPK-PPARα signal pathway

**Fig. 6:**
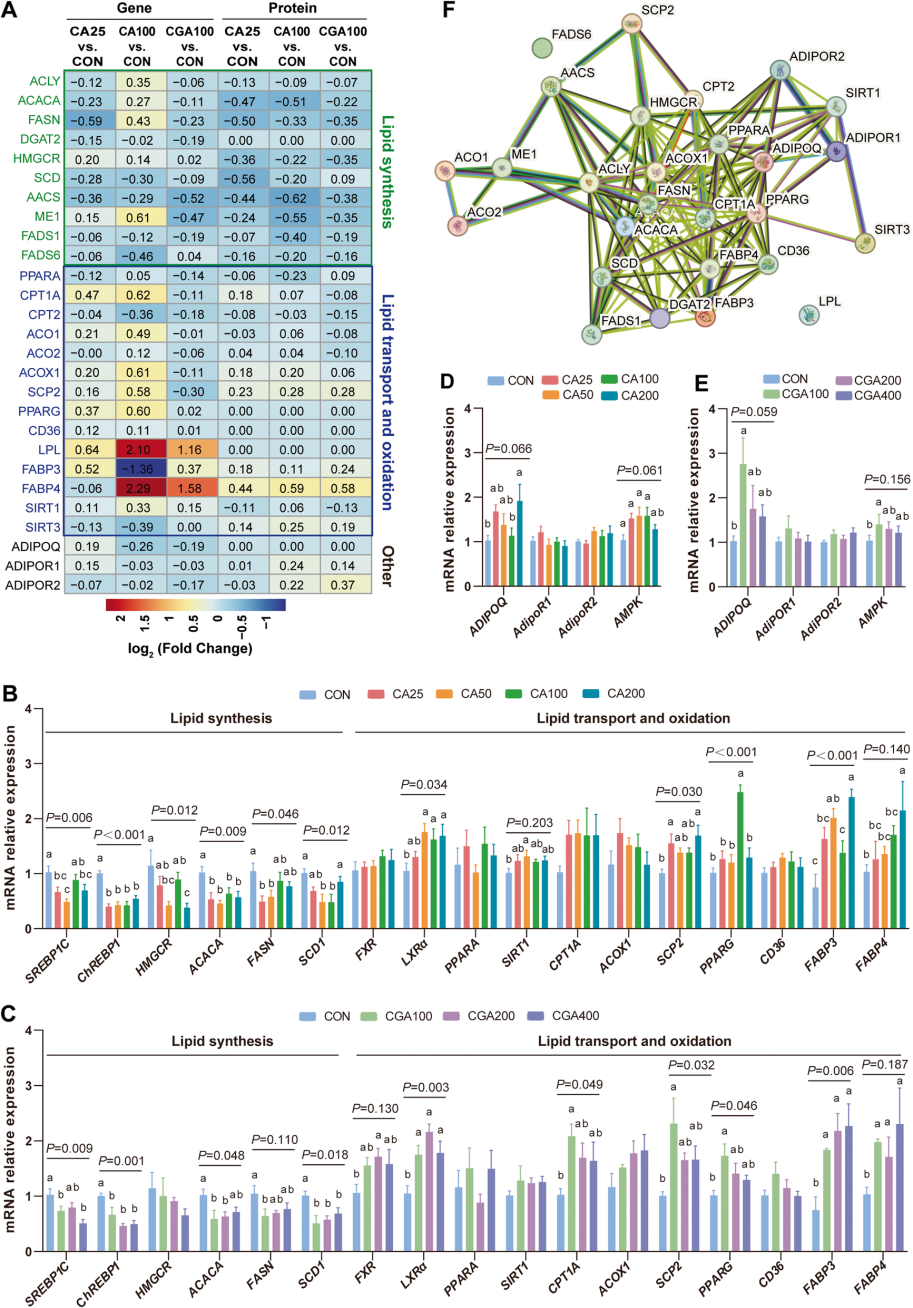
Comprehensive analysis of liver gene transcription and protein. **A** The relative genes transcription and protein expression levels of lipid synthesis, lipid transport and oxidation. **B–E** The relative mRNA expression levels of lipid synthesis, lipid transport and oxidation (*n* = 6). **F** Protein interaction analysis of target genes by STRING. ^a−c^Means with different letters differ significantly (*P* < 0.05)

As illustrated in Fig. 6A, joint transcriptomic and proteomic analysis revealed that genes involved in fatty acid biosynthesis (*ACLY*, *ACACA*, *FASN*, *HMGCR*, *SCD1*, and *ME1*) were downregulated following CA25 and CGA100 intervention. Conversely, genes associated with fatty acid oxidation and transport (*PPARA*, *PPARG*, *CPT1*, *CD36*, *LPL*, *FABP3*, and *FABP4*) were upregulated. These findings were corroborated by qRT-PCR analysis in CA and CGA treatment groups (Fig. 6B and C). Current studies have indicated that the activation of AMPK is crucial for the function of CGA [45, 46]. Additionally, adiponectin receptors AdipoR1 and AdipoR2 serve as upstream activators of AMPK and PPARα, respectively [47]. Interestingly, the genes expression of *AMPK*, *AdipoR1*, and *AdipoR2* were increased following CA and CGA treatment (Fig. 6D and E). Furthermore, protein interaction analyses showed strong relationships among these genes (Fig. 6F). Collectively, these results suggested that CA and CGA might reduce hepatic lipid accumulation through activation of the ADPN-AMPK-PPARα signaling pathway.

## Discussion

In the current study, we found that dietary CA and CGA can ameliorate hepatic lipid metabolism disorder and steatosis, thereby improving the egg production performance of laying hens.

Our findings suggest that laying hens in the late laying period showed typical symptoms of fatty liver in CON group, characterized by the emergence of yellow color and elevated hepatic lipid storage which are in line with previous literature [48, 49]. Here, we showed that CA and CGA ameliorated the typical symptoms of fatty liver when introduced into the diet. The incidence of fatty liver in laying hens adversely impacts both laying performance and egg quality, posing a threat to their health and productivity [2, 10]. Here, our results indicated that treatment with CA and CGA effectively improved the production performance and egg quality of laying hens. These therapeutic effects largely meet the recommended criteria for the treatment of fatty liver [50–52].

The pathological and physiological mechanisms underlying fatty liver in laying hens remain poorly understood. However, studies have shown similarities to mammalian MAFLD, all of which involve in lipid metabolism disorders, hepatic steatosis, inflammatory responses, and oxidative stress processes [53–56]. Dyslipidemia has been widely postulated to play a primary and fundamental role in the pathogenesis of fatty liver. It is usually characterized by elevated levels of TG, TC, VLDL-C and decreased levels of HDL-C [5, 57]. Notably, addressing dyslipidemia has been demonstrated to be effective in preventing or delaying the progression of fatty liver [58]. A previous report indicated that CA and CGA supplementation inhibits the progression of dyslipidemia in HFD-induced mice [59, 60], which showed similar effects in laying hens in our study that the administration of CA and CGA resulted in a significant reduction in serum levels of TG and TC. Plasma enzyme activities such as ALT and AST, which are released into the bloodstream upon hepatocyte injury, were employed to assess liver damage [44, 61]. As anticipated, we observed significant reductions in AST and ALT activities following the administration of CA25 and CGA100. Lipid metabolism disorder in the liver is a hallmark of fatty liver disease, leading to elevated TG and TC levels, and decreased HDL-C [55, 56]. Interestingly, Jia et al. [62] suggested that lipoprotein density correlates with cholesterol transport; HDL-C and VLDL-C are protective against hepatic lipid metabolism, whereas LDL-C is detrimental. Here, our results demonstrated that both CA25 and CGA100 significantly lowered TG and TC levels in hepatic tissues. Furthermore, our study revealed that LDL-C levels decreased in both the CA and CGA groups, and HDL-C levels increased significantly in the CA treatment group. Interestingly, we found that these effects did not exhibit a linear dose–response relationship; improvements were not significant when CA exceeded 25 mg/kg and remained non-additive when CGA exceeded 200 mg/kg. Interestingly, the egg laying performance of the CA25 group was better than that of the high-dose CA group. The liver is universally acknowledged as the pivotal organ for lipid biosynthesis and metabolism. In the context of oogenesis, yolk lipids are predominantly synthesized within hepatocytes. Impairments in hepatic metabolic function led to lipid accumulation, resulting in steatosis, which subsequently impedes vitellogenesis, thereby reducing both egg yield and quality. Our findings indicated that the transcriptomic analysis of lipid synthesis-related genes revealed a more pronounced down-regulation in the CA25 group, whereas certain lipid synthesis genes within the CA100 group exhibited up-regulation, notably including key enzymes such as ACLY, ACACA, and FASN. These results suggest that the CA100 regimen may be less effective than CA25 in mitigating fatty liver disease, which may explain why the egg production performance of the CA25 group is better than that of the high-dose CA treatment group.

Persistent lipid metabolism disorders can induce liver lipid peroxidation and subsequently lead to ROS accumulation [63, 64], resulting in diminished antioxidant capacity, culminating in oxidative damage to hepatic cells [61]. Laying hens with fatty liver usually exhibit elevated ROS and MDA contents and significantly lowered activities of GSH-PX, T-SOD, and T-AOC [65, 66]. Our study demonstrated that CA25 and CA50 additives increased the activity of GSH-PX, but notably increased MDA content in serum. Nonetheless, dietary CA and CGA both significantly enhanced antioxidant activity of T-SOD in liver, indicating a general positive effect of CA and CGA administration on liver antioxidant capacity.

CA and CGA (catechin and esters of quinic acid) share similar structures and pharmacological actions [67, 68]. CGA can be hydrolyzed into CA, which is one of the major active metabolites of CGA both in vivo and in vitro [69, 70]. On average, CGA absorption is 33%, while CA absorption is as high as 95% in rats or humans [33, 34]. Our study demonstrated that one-fourth the dosage of CA (25 mg/kg) exerted an equivalent improvement effect on laying hens compared to CGA (100 mg/kg). Based on these findings, we speculated that one-third, or even less, of CGA and almost all of CA were absorbed in the laying hens. This implies that only a small part of CGA enters into the bloodstream, with most being excreted in the stool. Research indicates that the absorption and utilization of CGA largely depend on its metabolism by colonic gut microflora in rats [32]. However, it is unclear whether the same is true for laying hens, especially given the underdeveloped colon in laying hens.

Liver transcriptomics and proteomics analysis revealed that fatty acid biosynthesis pathways were inhibited, whereas fatty acid transport and oxidation pathways were enhanced in our data. This finding aligns with the mechanism for treating fatty liver [66]. Hepatic lipogenesis is primarily regulated by the transcription factors of SREBP1 and ChREBP1, which activate downstream lipogenic genes, including *FASN* (the key enzyme for catalyzing fatty acid synthesis), *ACACA* (the rate-limiting enzyme for fatty acid synthesis), and *SCD1* (the major enzyme for catalyzing lipogenesis), thereby contributing to hepatic lipid accumulation [71–74]. Conversely, activation of the transcription factor *PPARA* (*PPARα*) promotes the expression of its downstream genes, such as *CPT1A*, *CD36*, *FABP3*, *FABP4*, and *LPL*, enhancing lipid transport and oxidative utilization in the liver [75]. Consistent with these findings, our results demonstrated that CA and CGA inhibited *SREBP1* and *ChREBP1*, and downregulated the gene expression of *ACLY*, *ACACA*, *FASN*, and *SCD1*. Additionally, *PPARα*, along with its downstream target genes (*CPT1A*, *CD36*, *FABP3*, *FABP4*, and *LPL*), were upregulated. Studies have shown that the FXR/LXRα receptor is a crucial mediator in the liver, up-regulation of FXR and down-regulation of LXRα can alleviate high-fat diet-induced MAFLD [76, 77]. In line with literature, our results exhibited that *FXR* and *LXRα* were up-regulated simultaneously. These findings collectively suggest that the effect of CA and CGA against fatty liver may be attributed to diminishing lipid synthesis and accelerating fatty acid oxidation.

Recent studies have demonstrated that the AMPK signaling pathways play crucial regulatory roles in CGA-induced benefits [20, 45]. It was also found that activated *AMPK* suppresses the expression of *ACACA* and *FASN*, which may reduce fatty acid synthesis, serum triglycerides, and cholesterol levels, thereby alleviating hepatic steatosis [45]. Peroxisome proliferator-activated receptors (*PPARs*) are crucial in the pathogenesis of non-alcoholic fatty liver disease (NAFLD), *PPARα* primarily enhances fatty acid oxidation in the liver, while *PPARG* (*PPARγ*) facilitates lipid transport [78]. Here, our results revealed that CA and CGA promoted the mRNA expression of *AMPK* and *PPAR*α in liver of laying hens. In the current literature on the upstream activation proteins of AMPK and PPARα, it is well-established that adiponectin binds to adiponectin receptors AdipoR1 and AdipoR2 and exerts anti-lipid effects in obesity via activation of AMPK and PPARα pathways, respectively [47]. At present, there is no report on the regulation of adiponectin and its receptor AdipoR1/2 by CA and CGA. Our results observed that CA and CGA up-regulated adiponectin and its receptor AdipoR1/2, along with the up-regulation of *AMPK* and *PPARα*. According to these results, we initially inferred a potential role of adiponectin in the activation of the AMPK-PPARα pathway by CA and CGA. However, how CA and CGA regulate the ADPN-AMPK-PPARα pathway, especially the regulation of adiponectin and AdiporR1/2 by CA or CGA, still needs to be further clarified in vitro hepatocyte experiments.

Additionally, this study will offer theoretical insights into the efficient utilization of CGA (including its isomers, neochlorogenic acid and cryptochlorogenic acid). For instance, these compounds can be directionally transformed into CA through methods such as microbial enzymatic hydrolysis, in vitro hydrolysis, and chemical structural modification, thereby enhancing their utilization efficiency.

## Conclusions

Our study shows that CA and CGA can be two outstanding feed additives for the prevention and mitigation of fatty liver by regulating lipid synthesis and transport metabolism, and ameliorating hepatic lipid metabolism disorder and steatosis. Moreover, CA may be one of the principal active metabolites of CGA in laying hens. CA also represents a more economical and efficient additive for alleviating fatty liver compared to CGA. Our work provides foundational evidence for the potential therapeutic efficacy of CA and CGA in treating fatty liver and related metabolic disorders in laying hens via the activation of the ADPN-AMPK-PPARα pathway, and offers insights into the efficient utilization of CGA through targeted modification to CA.

## Supplementary Information

Additional file 1: Table S1 Statistics of transcriptome sequencing. Table S2–S4 Differentially expressed genes between CA25 group, CA100 group, CGA1000 group and CON group. Table S5–S7 Top 10–15 GO terms of DEGs between CA25 group, CA100 group, CGA1000 group and CON group. Table S8–S10 Top 20 KEGG pathways of DEGs between CA25 group, CA100 group, CGA1000 group and CON group. Table S11–S13 Differentially expressed proteins between CA25 group, CA100 group, CGA1000 group and CON group. Table S14–S16 Top 10–15 GO terms of differentially expressed proteins between CA25 group, CA100 group, CGA1000 group and CON group. Table S17–S19 KEGG enrichment analysis of DEPs between CA25 group, CA100 group, CGA1000 group and CON group. Table S20 GO terms related to lipid metabolism by Gene Set Enrichment Analysis. Table S21 KEGG pathways related to lipid metabolism by Gene Set Enrichment Analysis.

## Data Availability

The raw sequencing data have been deposited in the China National Center for Bioinformation (CNCB) of BIG Submission Portal (BIG Sub) with the accession number CRA020037 (transcriptome) and OMIX007802 (proteome).

## Abbreviations

ACACA: Acetyl-CoA carboxylase
ACOX1: Acyl-CoA oxidase 1
ADFI: Average daily feed intake
ADPN: Adiponectin
ALT: Alanine aminotransferase
AMPK: AMP-activated protein kinase
AST: Aspartate aminotransferase
CA: Caffeic acid
CGA: Chlorogenic acid
ChREBP1: Carbohydrate response element binding protein 1
CoA: Coenzyme A
CON: Control
CPT1A: Carnitine palmitoyl transferase 1A
DEG: Differentially expressed gene
DEP: Differentially expressed protein
FABP3: Fatty acid binding protein 3
FABP4: Fatty acid binding protein 4
FASN: Fatty acid synthase
FCR: Feed conversion rate
GO: Gene Ontology
GSH-PX: Glutathione peroxidase
HDL-C: High-density lipoprotein cholesterol
H&E: Hematoxylin-eosin
KEGG: Kyoto Encyclopedia of Genes and Genomes
LDL-C: Low-density lipoprotein cholesterol
LPL: Lipoprotein lipase
MAFLD: Metabolically associated fatty liver disease
MDA: Malondialdehyde
ME1: Malic enzyme 1
PCA: Principal component analysis
PPARA/PPARα: Peroxisome proliferator-activated receptor α
PPARG /PPARγ: Peroxisome proliferator-activated receptor γ
ROS: Reactive oxygen species
SCD1: Stearoyl-CoA desaturase 1
SREBP-1c: Sterol regulatory element binding proteins-1c
T-AOC: Total antioxidant capacity
TC: Total cholesterol
TG: Triglyceride
T-SOD: Total superoxide dismutase
VLDL-C: Very low-density lipoprotein cholesterol
